# Metal Levels in Blood of Three Species of Shorebirds during Stopover on Delaware Bay Reflect Levels in Their Food, Horseshoe Crab Eggs

**DOI:** 10.3390/toxics5030020

**Published:** 2017-08-28

**Authors:** Joanna Burger, Nellie Tsipoura, Michael Gochfeld

**Affiliations:** 1 Division of Life Sciences, Rutgers University, 604 Allison Road, Piscataway, NJ 08854, USA; 2 Environmental and Occupational Health Sciences Institute, Piscataway, NJ 08854, USA; mg930@eohsi.rutgers.edu; 3 New Jersey Audubon, 11 Hardscrabble Rd, Bernardsville, NJ 07924, USA; nellie.tsipoura@njaudubon.org; 4 Rutgers Robert Wood Johnson Medical School and School of Public Health, Piscataway, NJ 08854, USA

**Keywords:** arsenic, cadmium, chromium, lead, mercury, shorebirds, red knot, sanderling, sempalmated sandpiper, blood, feathers, horseshoe crab eggs, *Limulus polyphemus*

## Abstract

Understanding the relationship between metal level in predators and their prey is an important issue, and is usually difficult to determine because animals eat a variety of organisms. However, shorebirds that stop over during spring migration along Delaware Bay (New Jersey) stay for only 2–3 weeks, and eat mainly horseshoe crab (*Limulus polyphemus*) eggs. In this paper, we examine the relationship between metal levels in horseshoe crab eggs, and blood and feather levels of metals in red knot (*Calidris canutus rufa*; *n* = 30), sanderling (*Calidris alba*; *n* = 20) and semipalmated sandpiper (*Calidris pusilla*; *n* = 38) from Delaware Bay. There is a rich literature on metal levels in feathers. For all three species, the levels of arsenic, cadmium, chromium, lead and mercury in blood were highly correlated with the levels of metals in the eggs of horseshoe crab (17 pooled samples). This indicates that the levels in the blood of these shorebirds quickly reflect levels in their prey (horseshoe crab eggs), while metals in the feathers were not correlated with the levels in eggs. Semipalmated sandpipers had the lowest levels of arsenic in blood and the highest levels of arsenic in feathers, compared to the other species. At Delaware Bay, semipalmated sandpipers have a diet higher in marsh invertebrates than the other species, which may account for the differences. The levels of cadmium and chromium in blood were significantly higher in knots than other species; knots only ate horseshoe crab eggs. For all of the metals except arsenic, the ratio of levels in blood/feathers was similar among species. For arsenic, the ratio of levels in blood/feathers were significantly lower in semipalmated sandpipers than in the other species, by an order of magnitude.

## 1. Introduction

Government agencies, conservationists, and the public are interested in levels of heavy metals in wildlife that could prove detrimental to the organisms themselves, or to those that eat them. Heavy metals in biota can also be used to indicate information about the levels in the foods they eat. Coastal birds are often used as bioindicators of contamination because they live in a variety of habitats, occupy different trophic levels, and are exposed to a range of chemicals [1,2,3]. Further, levels are often higher in coastal areas because of the runoff from rivers [4,5], as well as atmospheric deposition [6,7]. Metals can be sequestered in bottom sediments of bays and estuaries, to be released by storms or strong tides.

Shorebirds are one of the groups of birds that are mainly coastal; most shorebird species use marine habitats at some time in their life cycle [8]. Many shorebirds migrate long distances from their Arctic breeding grounds to their wintering grounds in South America [9,10,11], stopping to refuel along the Atlantic coast [12]. Over the last 30 years, many species of shorebirds have declined sharply [13,14,15]. In the 1990s, about 20% of the world’s shorebirds were listed as species of special concern [16], and now Andres et al. [17] estimate that 61% of American shorebird populations have declined in the last 30 years. For some species, such as semipalmated sandpipers (*Calidris pusilla*), data indicate that population declines are mainly occurring in the Atlantic flyway and in the eastern Arctic breeding region [18]. These declines have partly been attributed to difficulty foraging during migration, especially on Delaware Bay [19]. Shorebirds are particularly vulnerable, because many breed in harsh Arctic conditions with a very short breeding season, migrate over long distances, and stop over to refuel at a very small number of coastal bays and estuaries. Thousands stop at the same time and place, making them vulnerable to predation, variable food supplies, intense competition, and human disturbance [20]. 

During a two to three week stop-over period at Delaware Bay, several species of shorebirds stop to refuel, mainly on the eggs of horseshoe crabs (*Limulus polyphemus*) [10,21,22]. Delaware Bay supports the largest concentration of northbound shorebirds on the East Coast [23], as well as the largest spawning concentration of horseshoe crabs [24]. Amplexing pairs of horseshoe crabs spawn in the sand at high tide, depositing their eggs in nests that are too deep for the birds to access [25,26,27]. When the crabs are abundant, newly arriving females dig up the nests of previous spawning females while they deposit their own eggs, releasing the earlier eggs to the surf, making them available to foraging shorebirds [10,11,28]. The shorebirds concentrate at the high tide line where the eggs can form dense mats, although they also forage on the intertidal habitat [29]. Decreases in the populations of horseshoe crabs (overharvested for eel and conch bait, Kraemer and Michels [30]) have resulted in decreased egg availability, which in turn has resulted in declines in the number of shorebirds on Delaware Bay [10,11,31,32].

One of the shorebirds that depends upon the eggs of horseshoe crabs during the stopover at Delaware Bay each spring is the red knot (*Calidris canutus rufa*), which is currently listed as threatened [32,33]. During the spring stopover at Delaware Bay, the red knots and other shorebirds nearly double their weight in order to successfully fly, non-stop, the 6500-km journey to their Arctic breeding grounds [34,35]. With increasing urbanization and industrialization along our coasts, species that forage almost exclusively along the coasts are also exposed to increasing levels of contaminants [36,37,38]. Thus, shorebirds are exposed to whatever contaminants are in the horseshoe crab eggs, and they eat thousands while in the Bay [22,39]. 

Red knots and semipalmated sandpipers spend a great deal of time along Delaware Bay foraging on the eggs of horseshoe crabs each May, and although sanderling (*Calidris alba*) also forage by the thousands along the Bay, they also spend more time foraging along the Atlantic coast beaches (where very few horseshoe crabs spawn) than the other species. Heavy metal concentrations in the blood of knots and semipalmated sandpipers are correlated with the levels of arsenic, cadmium, chromium, lead, and mercury in horseshoe crab eggs [3]. In this paper, we tested the hypothesis that metal levels in the blood of sanderling were also correlated with those in the eggs of horseshoe crabs, and whether the relationships would be similar to the other two species. We were also interested in whether there was the same ratio of metal levels in blood and feathers for all three species. 

## 2. Materials and Methods

**Figure 1:**
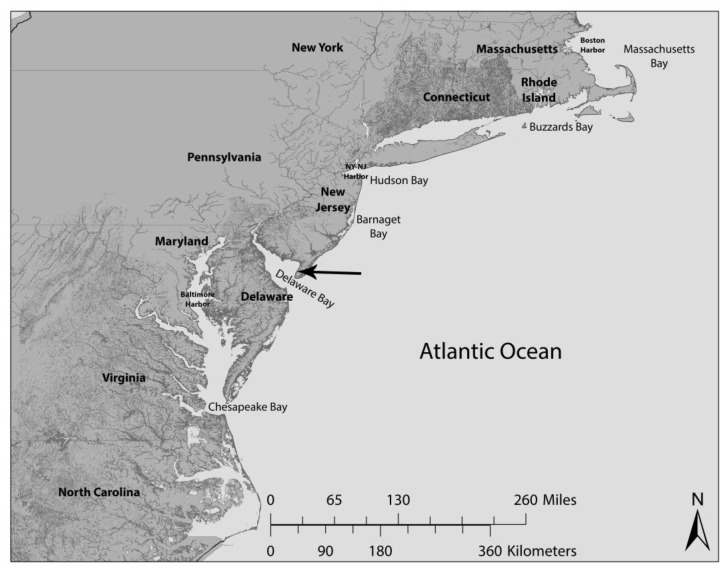
Map showing location of collection of blood of shorebirds and the eggs of horseshoe crabs (see arrow) along Delaware Bay.

Our overall protocol was to collect eggs of horseshoe crabs and to collect blood from shorebirds [37,38] from Delaware Bay, under appropriate permits (Figure 1). Eggs were collected from recently laid horseshoe crab nests on spawning beaches in 2012 [39]. Eggs were collected from recently laid clutches, placed in plastic bags, and frozen for later analysis [39]. We pooled eggs from several nests, and analyzed 17 pooled samples. Shorebirds were captured by cannon net. Blood was collected from shorebirds in 2011 and 2012, and placed in vials for later analysis at the Environmental and Occupational Health Sciences Institute of Rutgers University. We collected approximately 10 µL of blood in 75-uL heparinized capillary tubes. Tubes were placed in labeled non-additive glass Vacutainers to prevent breakage, placed upright on ice, and frozen the same day for later analysis [38]. Blood was collected 4 days after arrival to give the birds time enough to begin restoring weight lost during migration. Blood levels did not vary among years, and the data were pooled. We pulled about 25 breast feathers/shorebird, and stored them in plastic envelopes [3]. Blood and feather samples were not pooled across birds. Individual results were obtained for 30 red knots, 20 sanderlings, and 38 semipalmated sandpipers. All methods were approved by the Rutgers University IACUC (92-036), and conform to guidelines provided by the Ornithological Council (www.nmnh.si.edu/BIRDNET/GuideToUse). These guidelines have been formulated with consideration of animal welfare and research needs. 

Horseshoe crab eggs and avian blood samples were kept frozen until they were transferred to the Elemental Analysis Laboratory of the Environmental and Occupational Health Sciences Institute at Rutgers University. Total mercury was analyzed by cold vapor atomic absorption spectrophotometry, of which about 85–90% is assumed to be methylmercury [40]. Other metals were analyzed by flameless, graphite furnace atomic absorption. Instrument detection limits were 0.02 ppb for arsenic and cadmium, 0.08 ppb for chromium, 0.15 ppb for lead, and 0.2 ppb for mercury. All specimens were analyzed in batches with known standards, calibration standards, and spiked specimens. Blanks, standard calibration curves, and spiked matrix specimens were used to monitor assay performance for all batches. All concentrations are expressed in ppb (ng/g), wet weight for total metal. Recoveries ranged from 87% to 101%. Batches with recoveries of less than 85% were reanalyzed. The coefficient of variation on replicate, spiked samples ranged up to 10%. 

We used non-parametric procedures (Kruskal Wallis test, PROC NPAR1WAY [41]) to determine species-related differences in heavy metal levels, and Kendall tau correlations for determining relationships between horseshoe crab egg levels and shorebird blood levels. We used these non-parametric tests because they are more conservative and are best fitted for small datasets.

## 3. Results

There were significant interspecies differences in the arsenic levels in both blood and feathers (Table 1). Semipalmated sandpipers had the lowest levels of arsenic in their blood, but the highest in their feathers. There were significant interspecific differences in the levels of cadmium in blood (but not feathers); red knots had the highest levels of cadmium compared to the other species. Levels of lead in blood, and mercury in feathers, almost reached interspecific significance, and might have with larger sample sizes (Table 1). The ratio of metals in blood/feathers is of interest because it might indicate that levels in either blood or feathers might be useful as a bioindicator if the ratios were similar among species. However, the ratios differed significantly for arsenic and chromium because the levels in blood differed significantly (Table 1). 

**Figure 2:**
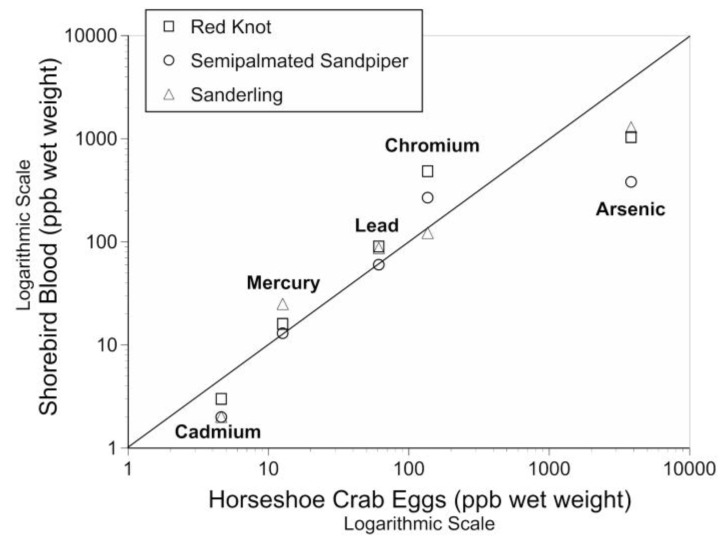
Relationship of mean levels of arsenic, cadmium, chromium, lead and mercury in the blood of three species of shorebirds and the mean level in eggs of horseshoe crabs, their primary prey while on migratory stopover in May at Delaware Bay, New Jersey. (See Table 1).

For all three species, the mean metal levels in the blood were positively correlated with the mean levels of metals in the eggs of horseshoe crabs, their primary prey at Delaware Bay in the spring (Figure 2). For all three species, the Kendall tau was 1 (*p* < 0.01) as they were completely aligned. Thus, the hypothesis that the metal levels in the blood of sanderling reflected those of crab eggs, and were similar to those of knots and semipalmated sandpipers was supported. The mean levels of cadmium and arsenic in blood of shorebirds were slightly below levels in horseshoe crab eggs, and arsenic was especially lower for semipalmated sandpipers (Figure 2). 

## 4. Discussion

### 4.1. Relationship between Metals in Prey and in Shorebird Blood

The levels of heavy metals in the blood of all three species were positively correlated with the levels in horseshoe crab eggs, their primary food while they are in Delaware Bay. Although the levels in their prey (horseshoe crab eggs) were slightly lower for some metals, they were the same order of magnitude. These data indicate that the blood quickly reflects local prey levels [3,5]. While many studies assume that heavy metal levels in blood reflect local food levels [42,43], this is usually difficult to show because most birds eat many different prey items. In contrast, in Delaware Bay, the shorebirds primarily eat horseshoe crab eggs [22], and eat more than their normal diet, as they have to gain sufficient weight for the long migratory journey [34,35]. This may lead to higher accumulation of metals in the blood, because they are processing higher quantities of horseshoe crab eggs than they normally would. This suggests that it would be interesting to examine the levels of metals in blood of a local resident shorebird or gull (e.g., laughing gull, *Leucophaeus atricilla*) that eats horseshoe crab eggs (but does not need to nearly double its weight in 2–3 weeks) during the spawning period, and again in the summer, when horseshoe crab eggs are no longer available.

Geographical data on horseshoe crab eggs from other places along the Atlantic coast indicate that arsenic levels are lower in Delaware Bay than most other places from Maine to Florida, while the levels of the other metals are similar among regions [44]. However, the levels of arsenic in horseshoe crab eggs from farther north are 2–3 times higher than those in Delaware Bay, indicating that exposure for shorebirds would be higher if they ate crab eggs farther north. Over time, arsenic has remained relatively constant in horseshoe crab eggs in Delaware Bay, while cadmium and mercury have declined, and chromium and lead showed no clear pattern [39]. 

Many species of shorebirds, including red knots, engage in multiday non-stop flights, consuming not only fat, but organ tissue as well. Thus, during the first two or three days at a stopover site, they consume eggs rapidly to rebuild their body systems. Thereafter, the eggs contribute to the fat build-up necessary for migration. Red knots, for example, need to almost double their weight to successfully migrate to their northern breeding grounds and arrive with sufficient resources to breed [35]. Ingested heavy metals are absorbed from the gastrointestinal tract into the circulating blood, and then distributed to other organs with eventual excretion or storage, including the feathers. In general, no prey source other than horseshoe crab eggs contributes significantly to the circulating levels of heavy metals. The metal levels in the eggs of horseshoe crabs clearly and dramatically declined between 1994 and 2012, and presumably the decline has continued [39]. However, there has not been a corresponding consistent decline in metal levels in the feathers of the same shorebirds. The declines varied by species [37]. This difference is due to the fact that feathers reflect circulating blood levels at the time of feather formation (43), and the feathers were formed in South America [5] prior to northbound migration. Thus, metals were sequestered in feathers while they were growing, when the birds were in South America. After feather formation is complete, there is no more blood supply to the feather. The three species do not winter in the same areas of South America, although there is some overlap. The levels of metals in blood when the shorebirds first arrive at Delaware Bay reflects mobilization from tissues during migration. If shorebirds lingered in Florida or farther south, blood levels would reflect levels in foods obtained at a more southern stopping area. For this reason, all blood was collected more than 4 days after the arrival of the shorebirds on Delaware Bay.

### 4.2. Species Differences

The levels of heavy metals in the blood of the three shorebirds showed similar patterns; levels reflected those in the eggs of horseshoe crabs (their prey). However, there were interspecific differences in arsenic for both blood and feathers, and for cadmium and chromium in blood. Semipalmated sandpipers had lower levels of arsenic in blood, and higher levels of arsenic in their feathers than the other species. The blood of shorebirds also had higher levels of arsenic than the other metals, which clearly reflects high levels in the eggs of horseshoe crabs [39]. Further, the blood levels of cadmium and chromium were higher in red knots than the other species. The reasons for these species differences are unclear, but might reflect initial differences when birds arrived at the bay, or food differences. Semipalmated sandpipers have a broader diet than other species, and consume marsh invertebrates as well as eggs [22,45], while the other species do not generally feed in marshes. Red knots had significantly higher levels of cadmium and chromium, which might reflect their complete reliance on horseshoe crab eggs, while sanderling sometimes feed along the Atlantic shore on invertebrates.

There were no interspecific differences in lead and mercury, two of the contaminants of main concern for species foraging in coastal and marine habitats. These two metals can cause a variety of adverse effects at higher levels (reviewed generally in Eisler [46,47] and Burger and Gochfeld [5]) and for these shorebirds, Tsipoura et al. [38]. Levels were generally below toxic effects level [38].

### 4.3. Ratio of Metal Levels in Blood/Feathers for Shorebirds

There are more studies of metal levels in feathers of birds than any other tissue, partly because heavy metals (e.g., mercury) concentrate in feathers, feathers are easy to collect non-destructively, can be stored easily, and the large number of studies with feathers allows comparisons among species and regions [3,48,49,50]. Feathers reflect blood levels of metals at the time they were grown [5]. Conversely, metals are not mobilized from feathers, so those metals do not contribute to toxicity. Thus, it is useful to know whether the same relationship exists between levels in feather and blood, because it indicates something about uptake of the metals in blood, and suggests whether the birds were coming from areas with the same contamination as obtained from Delaware Bay, or not. Arctic-breeding shorebirds usually grow their feathers on the wintering grounds or during northward migration [51,52]. Thus, because the three species examined spend the winter in different places (where the feathers were grown), it is not likely that the ratios would be the same among the three species. We found that ratios were inconsistent, reflecting the difference in diet during molt (in South America) vs while the shorebirds were in Delaware Bay.

## 5. Conclusions

All three species of shorebirds showed a similar, positive correlation of the levels of heavy metals in their blood with the levels of heavy metals in their primary prey (horseshoe crab eggs) while at the migratory stopover on Delaware Bay. The shorebirds had been in Delaware Bay more than 4 days, but less than 2 weeks, when blood was taken, indicating that within this time period, their blood levels reflect those in horseshoe crab eggs. This study provides clear evidence that levels of metals in the blood of shorebirds are closely correlated with those in their prey, and the relationship occurs rapidly (within days of arrival). Red knots had significantly higher levels of cadmium and chromium in their blood than the other species, reflecting their greater reliance on horseshoe crab eggs in their diet. 

## Figures and Tables

**Table 1:** Levels of heavy metals (mean ± SE, ppb) in shorebirds collected at Delaware Bay, New Jersey, for blood, feathers, and the ratio of blood/feathers.

	Arsenic	Cadmium	Chromium	Lead	Mercury
Red Knot					
Blood (*n* = 30)	1036 ± 188	2.9 ± 0.7	484 ± 62	90 ± 12	16 ± 3.1
Feather (*n* = 30)	446 ± 42	17 ± 2.4	578 ± 83	484 ± 67	576 ± 105
Ratio	2.32	0.17	0.84	0.19	0.03
Sanderling					
Blood (*n* = 20)	1288 ± 193	2 ± 0.7	122 ± 21	87 ± 14	25 ± 5.3
Feather (*n* = 20)	311 ± 64	10.5 ± 2.6	463 ± 63	367 ± 52	730 ± 109
Ratio	4.14	0.19	0.26	0.24	0.03
Semipalmated sandpiper					
Blood (*n* = 38)	381 ± 45	1.8 ± 0.5	268 ± 52	59.8 ± 11	12.7 ± 3.3
Feather (*n* = 30)	842 ± 101	14.2 ± 2.7	524 ± 64	411 ± 46	428 ± 58
Ratio	0.45	0.13	0.51	0.15	0.03
Interspecific Differences (X^2^)					
Blood	31.9 (<0.0001)	6.7 (0.04)	27.9 (<0.0001)	5.0 (0.08)	3.0 (NS)
Feathers	25.4 (<0.0001)	2.9 (NS)	0.7 (NS)	0.8 (NS)	5.1 (0.08)
Ratio	37.9 (<0.0001)	1.4 (NS)	13.8 (0.001)	3.4 (NS)	0.09 (NS)
